# Results of an exploratory survey within ESTS membership in 2022 on current trend of robotic-assisted thoracic surgery and its training perspectives

**DOI:** 10.1093/icvts/ivae031

**Published:** 2024-03-05

**Authors:** Shilpa Gandhi, Nuria Maria Novoa Valentin, Alessandro Brunelli, Isabelle Schmitt-Opitz, Marialuisa Lugaresi, Niccolò Daddi, Herbert Decaluwe, Hasan Batirel, Giulia Veronesi, Jean-Marc Baste, Paraskevas Lyberis, Joel Dunning, ESTS Robotic Working Group, Nuria Maria Novoa, Alessandro Brunelli, Isabelle Opitz, Niccolò Daddi, Herbert Decaluwe, Hasan Batirel, Giulia Veronesi, Jean-Marc Baste, Paraskevas Lyberis

**Affiliations:** Cardiac surgery unit, Department of Cardiothoracic Surgery, St Georges’ University Hospital NHS Foundation Trust, London, UK; Division of Thoracic Surgery, Puerta de Hierro University Hospital, Majadahonda, Spain; Division of Thoracic Surgery, St James’s University Hospital, Leeds, UK; Department of Thoracic Surgery, Universitatsspital, Zurich, Switzerland; Department of Medical and Surgical Sciences, University of Bologna Medical School, Bologna, Italy; Division of Thoracic Surgery unit, IRCCS Azienda Ospedaliero-Universitaria, University of Bologna Medical School, Bologna, Italy; Division of Thoracic Surgery, University Hospital Leuven, Leuven, Belgium; Division of Thoracic Surgery, Biruni University School of Medicine, Istanbul, Turkey; Division of Thoracic Surgery, Universita Vita e Salute San Raffaele, Milan, Italy; Cardiothoracic Department, Rouen University Hospital, Inserm U1096, UNIVRouen, Normandy, France; Division of Thoracic Surgery, University of Torino, Torino, Italy; Division of Cardiothoracic Surgery, James Cook University Hospital, Middlesbrough, UK

**Keywords:** Thoracic surgery, Robotic-assisted thoracic surgery, Training, Robotic curriculum, Simulation, Video-assisted thoracoscopic surgery, Conformite Europeenne

## Abstract

**OBJECTIVES:**

Robotic-assisted thoracic surgery (RATS) is increasingly used in our specialty. We surveyed European Society of Thoracic Surgeons membership with the objective to determine current status of robotic thoracic surgery practice including training perspectives.

**METHODS:**

A survey of 17 questions was rolled out with 1 surgeon per unit responses considered as acceptable.

**RESULTS:**

A total of 174 responses were obtained; 56% (97) were board-certified thoracic surgeons; 28% (49) were unit heads. Most responses came from Italy (20); 22% (38) had no robot in their institutions, 31% (54) had limited access and only 17% (30) had full access including proctoring. Da Vinci Xi was the commonest system in 56% (96) centres, 25% (41) of them had dual console in all systems, whereas RATS simulator was available only in half (51.18% or 87). Video-assisted thoracic surgery (VATS) was the most commonly adopted surgical approach in 81% of centres (139), followed by thoracotomy in 67% (115) and RATS in 36% (62); 39% spent their training time on robotic simulator for training, 51% on robotic wet/dry lab, which being no significantly different to 46–59% who had training on VATS platform. There was indeed huge overlap between simulator models or varieties usage; 52% (90) reported of robotic surgery not a part of training curriculum with no plans to introduce it in future. Overall, 51.5% (89) responded of VATS experience being helpful in robotic training in view of familiarity with minimally invasive surgery anatomical views and dissection; 71% (124) reported that future thoracic surgeons should be proficient in both VATS and RATS. Half of the respondents found no difference in earlier chest drain removal with either approach (90), 35% (60) reported no difference in postoperative pain and 49% (84) found no difference in hospital stay; 52% (90) observed better lymph node harvest by RATS.

**CONCLUSIONS:**

Survey concluded on a positive response with at least 71% (123) surgeons recommending to adopt robotics in future.

**Figure:**
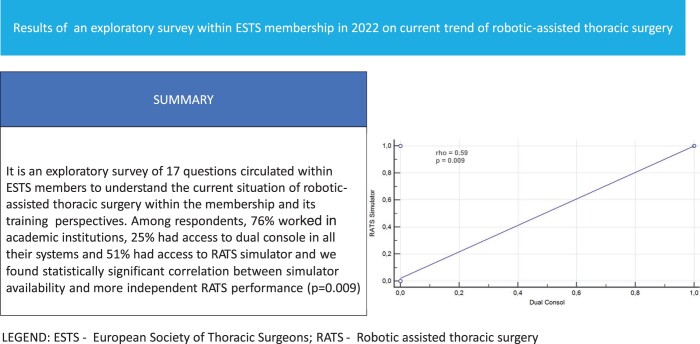

## INTRODUCTION

Minimally invasive thoracic surgery (MITS) has developed since 1990s, initially with pleural procedures but slowly expanding to Video-assisted thoracoscopic surgery (VATS) anatomical lung resections. The first description of VATS lobectomy with anatomical hilar dissection for cancer was published in 1992, followed by 1st reported series of robotic thoracoscopic surgery in 2002 [1]. After multiple published case series of successful robotic-assisted thoracic surgery (RATS) procedures, now slowly we are gaining definitive evidence of advantages of RATS over VATS [2]. However, there is a deficit in standardized curriculum or requirement for training residents in RATS. Until now, Intuitive Surgical Inc. (Sunnyvale, CA, USA) has delivered the only available robotic surgery platform [3]. Although training programme by Intuitive is divided into initial and advanced course, the success of it depends on many external factors [3]. Furthermore, the training is focused on experienced thoracic surgeons [3]. We have collated this article based on a survey created by the European society of thoracic surgeons (ESTS) Robotic Working Group to analyse the current practice of RATS across the ESTS worldwide membership with special reference to training and learning as a part of curriculum.

## METHODS

As per database, the total ESTS membership is 1790, which includes 29 honorary members and 67 senior members. This was an exploratory survey aiming to understand the current status of MITS practice with particulars to training perspectives amongst the members of ESTS. The training perspectives were outlined by—availability of robotic system (and its type) in a centre; accessibility to a dual console; accessibility to RATS simulator; time invested on a training platform for VATS or RATS or both. The study was based on a survey including 17 questions rolled out to all these ESTS members (Supplementary Material, Annex 1) via system-generated email. In the questionnaire, only 1 answer from each unit was accepted. On the basis of ESTS database and further crossing information between the demographics sections allowed us to avoid any duplications. The number of responses received was 10% as in previous surveys. As a consequence, 174 complete responses were received/included corresponding to 174 units (some skipped responding some questions). No ethical committee approval was necessary.

### Statistical analysis

Data were verified and subsequently imported into SPSS software version 15.0 for the analysis. The primary analysis was a descriptive summary, including calculation of frequencies, median and interquartile range. Spearman’s correlation index was adopted to assess the correlation between dual console and availability of RATS simulator with respect to training. The Chi-squared test or Fisher’s test (expected number <5) was used to analyse categorical variables, while the Mann–Whitney test was adopted to analyse continuous variables, for comparison between respondents from an academic or non-centre. A *P*-value <0.05 was considered significant. The statistical analyses results are as per the Supplementary Material, Annex 2.

## RESULTS

The total responses received were 174, inclusive of skipped responses; 84.39% (146) respondents were either board-certified or board-eligible (96—board-certified, 21—trainees and 56—chief of unit); 76% (128) belonged to academic institutions. A wide representation was observed as described in Table 1 with maximum participation from Italy (20).

**Table 1:** Demographics of survey participation on world map with countries and their corresponding respondents

Regions worldwide within ESTS	Countries with corresponding units/centres participating in the survey
Europe with UK/Ireland region (139)	Italy: 20, Germany: 14, Spain: 11, Switzerland: 10, France: 9, Belgium: 8, Austria: 3, Czech Republic: 2, Denmark: 3, Greece: 6, Hungary: 4, Netherlands: 7, Poland: 2, Portugal: 2, Sweden: 2, Turkey: 5, UK: 8, Ukraine: 2, Ireland: 1, Luxembourg: 1, Finland: 1, Belarus: 1, Macedonia: 1, Moldova: 1, Norway: 3, Lithuania: 1
Asian region (12)	Japan: 7, India: 3, Rep of Korea: 1, Malaysia: 1
Middle-East region (4)	Saudi Arabia: 1, Israel: 1, Jordan: 1
African region (2)	Algeria: 1, Morocco: 1
USA/Canada (22)	USA: 8, Brazil: 8, Canada: 3, Mexico: 2, Argentina: 1
Australia (2)	

ESTS: European society of thoracic surgeons.

**Figure 1:**
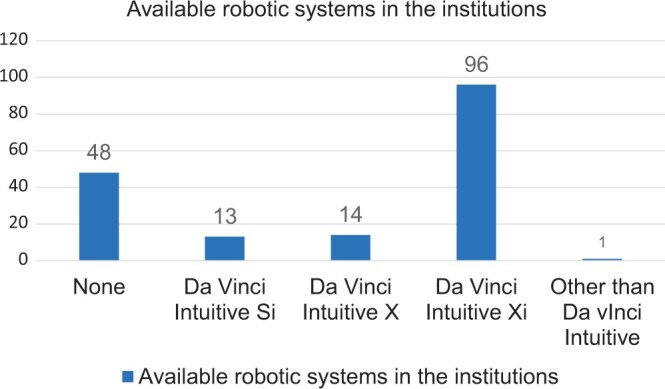
Figure demonstrating types of robotic systems available in the institutions across the European Society of Thoracic Surgeons.

**Figure 2:**
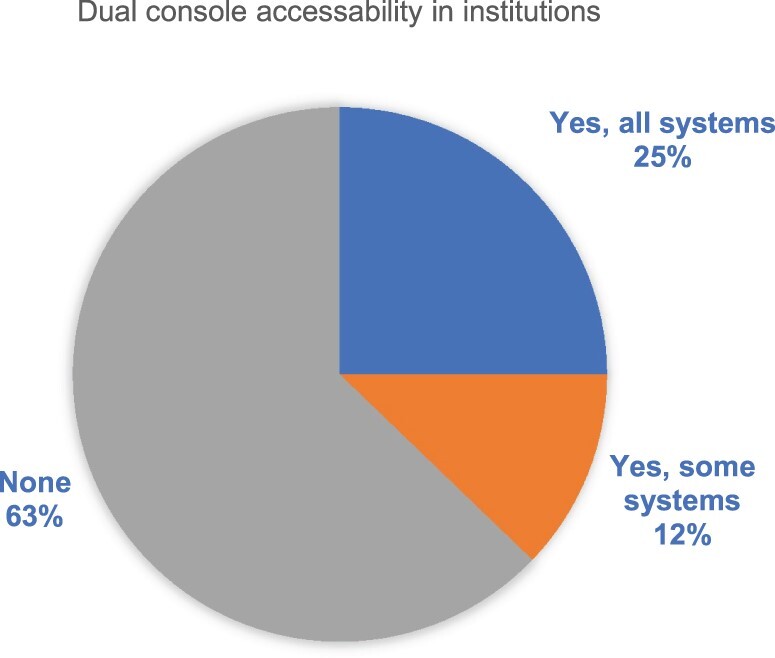
Figure demonstrating dual console accessibility in the robotic systems.

Ninety-six centres (56%) had Da Vinci Xi system in their institution (Fig. 1) with 41 having dual console (25%) (Fig. 2); 51% (87) had access to RATS simulator. Video-assisted thoracic surgery (VATS) was the most commonly adopted surgical approach in 139 centres, followed by thoracotomy (115) and RATS (62).

**Figure 3:**
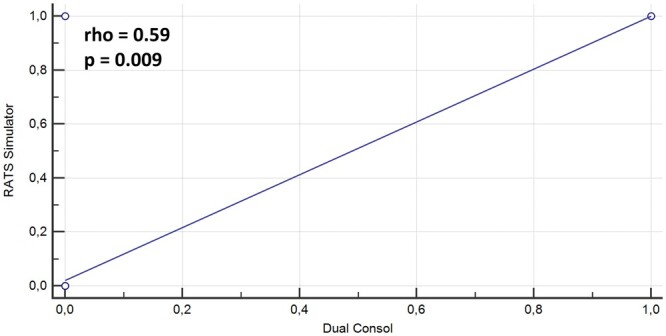
Scatter plot graph showing the direct relationship and the Spearman’s correlation index between dual console and availability of RATS simulator in trainee surgeons. RATS: robotic-assisted thoracic surgery.

**Figure 4:**
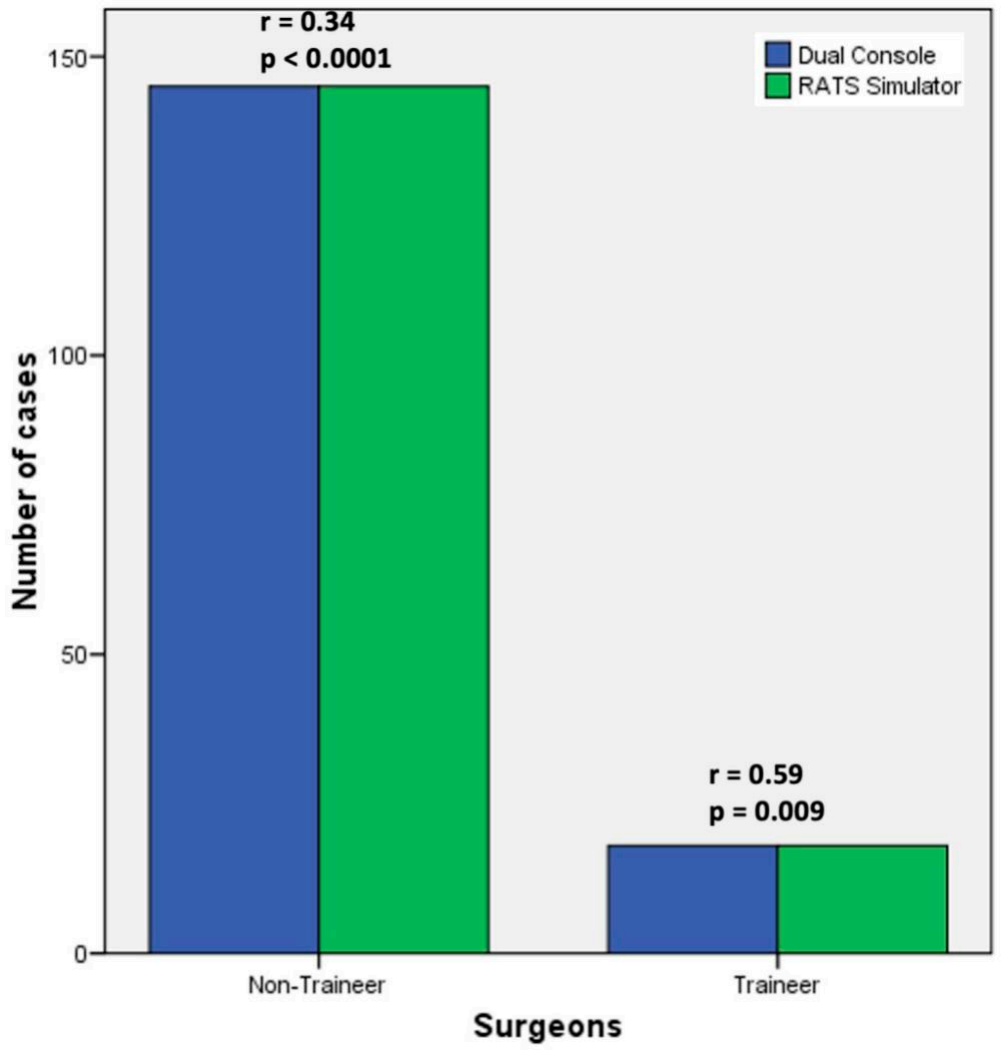
Bar graph chart showing Spearman’s correlation index between dual console and availability of RATS simulator with respect to training and independent robotic performers. RATS: robotic-assisted thoracic surgery.

Fifty-four respondents including chiefs and attending surgeons (other than chief), 5 trainees/fellow had an access to robot limited to <50% of all their lung resections and 30 respondents had full access including proctoring. A statistically significant correlation (rho = 0.59, *P* = 0.009) was found between dual console and availability of RATS with training and independent robotic performers (Figs 3 and 4). MITS practice observation identified 3 main groups as—VATS, RATS and mixed group (VATS and RATS), and results indicated direct correlation between dual console, RATS simulator and independent performers. We observed in our survey that 5–9% respondents engaged their training time in full across any training platform, one-third engaged <25% of their training time on it and another two-thirds on robotic simulator and wet/dry lab.

Twenty-nine centres adopted VATS as their exclusive surgical approach, of which 24 reported to be performing >50% anatomical lung resection. RATS as exclusive approach was incorporated in 6 centres with >80% anatomical lung resections being performed independently and spending 50–100% of their training time on RATS training platform (simulator, wet/dry lab). Overall, 81% responded to be major VATS performers for their anatomical lung resections, 5.73% performed <20% and 19% performed >80% of their surgeries by VATS. Even though 62 units adopted RATS for their resections, 52% of all 174 respondents admitted to have no plans to introduce RATS in their future curriculum, for unknown reasons. A total of 52% thought there was no difference in chest drain removal timing, 37% reported no difference in postoperative pain and 49% observed no difference in length of hospital stay; 52% preferred RATS for better lymph node harvesting. The above observations were attributed taking into consideration that all the responses obtained were devoid of any bias and irrespective of any seniority amongst the respondents. In any of the possibilities, duplication has been ruled out already.

## DISCUSSION

### Demographics

We received maximum responses from Italy (20) followed by Germany (14) and Spain (11). This likely reflects the membership numerosity and general interest for this subject across Europe.

### Robotic armamentarium

Worldwide, the da Vinci Si system has been available since 2009, 3rd generation da Vinci X was introduced in 2015. By December 2022, Intuitive reached 12 million procedures across the globe including all specialties [4]. Now non-Intuitive robots along with Da Vinci single-port robots are gaining momentum in our day-to-day practice.

There is enough emphasis on dual console for robotic training and education. Dual console facilitates teaching by allowing the teacher to switch from observer to operator, if needed, in a relatively seamless way [5]. Most importantly, the use of dual console formalizes the educational aspect of operation and is safe with equivalent outcomes [5]. This survey demonstrated a moderate statistically significant correlation (rho = 0.59, *P* = 0.009) between dual console and availability of RATS simulator with respect to training and surgeons performing independent robotic anatomical lung resections (Figs 3 and 4). This is valuable information stressing the importance of training essentials for RATS development in a centre.

### Robotic training and simulation

Shahin *et al.* have already published in their article the importance of dual console and simulator as integral for training in RATS. They also mentioned that despite having considerable availability of proctors in Europe (including UK), their availability and ability to commit to thorough training may vary due to their workload [3]. Use of da Vinci skill simulator, e-learning modules and plastic model has gained esteemed significance for skill enhancement for aspiring to be a console surgeon in RATS [3].

Another study reported the use of simulator directly contributing to significant skill and performance improvement compared to training using the surgical system on inanimate objects [5]. While the simulator has been shown to be an effective tool, there still lies an inherent leap of faith when moving from the simulator to a live patient [5]. Kindheart Simulator with animal model imparts a more realistic surgical experience compared to these simulations [3].

In our survey, we found 36% of respondents, irrespective of their stage in training or practice, stating that they spent at least 25% of their training time on any training platform, another two-thirds on robotic simulator and wet/dry lab. Only 5–9% submerged in full immersion off their training time.

#### Minimally invasive thoracic surgery practice observed

Further detailed analysis among 3 MITS access groups (VATS, RATS and mixed VATS + RATS) revealed that at least 18 respondents admitted to have not performed any anatomical lung resections independently, which included 10 surgeons/chief and 7 trainee/fellow/other. Interestingly, 3 of these centres reported to have been performing all lung resections by thoracotomy, 6 centres by VATS for >50% lung resections. Seven centres used RATS for <50% lung resections and spent less of their training time on any training platform. Overall, it appears, this group indulged in less training time.

Exclusive VATS as a surgical approach was used in 29 centres, whereby 24 of them reported to have been performing >50% anatomical lung resection with VATS. Fourteen out of these 24 centres indulged in spending some time (<25%) on VATS wet lab and only 1 used it for half of their time. Four centres indulged in training on VATS simulator for 50–75% of their time.

RATS as exclusive approach was used in 6 centres who performed >80% anatomical lung resections independently by RATS; of them, 4 centres spent 50–100% time on RATS training platform (simulator, wet/dry lab). In an overview, 24 centres have been performing >50% anatomical lung resections by RATS where 7 of them are involved in >50% of their time on robotic simulation. This further establishes the fact that simulation is an important factor in development of RATS skills in thoracic practice.

Fifty-three respondents used both VATS and RATS (irrespective of thoracotomy), where 14 used robot for >50% of anatomical lung resections and participating in simulator and wet/dry lab for 50–100% time (Table 2). It appears that centres with most RATS + VATS adoption indulged in extensive training on respective platforms (>50% time) thereby influencing the development of that subspeciality (especially RATS). Further, as per Supplementary Material, Annex 2 and Figs 3 and 4, it is already established that availability of dual console and a RATS simulator in an academic unit has significantly and directly contributed to increasing number of surgeons performing independent RATS.

**Table 2:** Graph depicting the total major performers with respect to surgical approach, in-training status and at least 50% or more time spent on robotic-assisted thoracic surgery simulator

Approach	*n*	Major performers (>50% surgeries)	In-training	>50% time on RATS simulator
Open thoracotomy	115	35	6	5
VATS	139	106	13	17
RATS	62	25	1	12
Mixed (VATS + RATS)	53	–	1	1

RATS: robotic-assisted thoracic surgery; VATS: video-assisted thoracic surgery.

### Teaching and curriculum

In 2017, the American Association for Thoracic Surgery published a proposed definition and nomenclature for robotic thoracic surgery and suggested robotic portal lobectomy or robotic pulmonary surgery as appropriate terms. The nomenclature system gathers information about the type of resection, necessity of an assist port, number of ports used and if the procedure met the definition of RATS [6]. The objective was to enable future adequate comparison between studies [6, 7]. Hence there is a need of formalized, detailed thoracic surgery-specific curriculum to be made available for training programmes to adopt easily. The University of Southern California has developed a stratified robotic curriculum on the basis of graded responsibilities within operation allowing efficient advancement of each one’s competencies as well [7]. In our survey, a large proportion of respondents (52%) admitted to have no plans to introduce RATS in their future curriculum for unknown reasons. Nevertheless, 13 centres from the USA, Belgium, Australia, France, Japan and Netherlands already had robotics into their training programme.

### The learning curve

Cerfolio *et al*. has established the effectiveness of their RATS training system for residents. He divided the lobectomy procedure into 19 steps involving different technical manoeuvres and evaluating resident performance through a score from 0 to 100%. Eventually, some residents delivered 90–100% performance achieving a stable patient outcome of major morbidity, mortality and a reduction in conversion to thoracotomy and major vascular injury [7]. This success was demonstrated elsewhere too in 100 robotic cases with no difference in operative outcomes when trainees were the primary surgeon [5].

We identified 139 (81.29%) respondents as major VATS performers for their anatomical lung resections: 5.73% performed <20% and 19% performed >80% of their surgeries. A total of 62 respondents considered themselves independent robotic surgeons but in 2019 (pre-covid), 34% of them performed <10% of their anatomical lung resections using RATS. To summarize, we detected a large discrepancy between RATS and VATS adoption (Table 3).

**Table 3:** Region-wise summary of adoption of approach in current practice and preferences in future

Regions worldwide within ESTS	Adoption of VATS/RATS in current practice and future preferences
America	All have access to robot and dual console, hence major RATS performers Mexico, Argentina, Chile and Canada had limited access to robot and dual console. Hence, VATS was most adopted approach and hence preferred for future practice.
European region	Belgium, France, UK, major units of Italy and Spain, Switzerland, Bulgaria and Norway had access to robot and dual console. They were major RATS performers and preferred both VATS/RATS for future practice. Some units of Austria, Italy, Luxembourg, Hungary, Jordan and half of Switzerland centres were proponent of VATS in future. Greece had no access to robot and were adopting mixed approach but preferred both VATS and RATS in future.
Asian region	Malaysia and half of centres of Japan preferred VATS due to lack of robot. India and remaining centres of Japan, despite having access to robot, were adopting for only 10% of their anatomical resection.
Middle-East region	Only Israel had robotic access and dual console with RATS being preferred approach. Saudi Arabia had no robotic system and adopted VATS for day-to-day practice.
African region	Algeria had no robotic system and adopted thoracotomy for anatomical resection, but preferred both VATS and RATS for their future practice. Morocco had no robotic system and adopted VATS for day-to-day practice.
Australia	Out of the 2 centres, 1 of them already had an established RATS training curriculum and major RATS independent performers.

ESTS: European society of thoracic surgeons; RATS: robotic-assisted thoracic surgery; VATS: video-assisted thoracic surgery.

Arnold *et al.* utilized a cumulative sum analysis of operating time to identify a 22-case learning curve with mastery achieved after 63 cases. However, this study acknowledged that the operating surgeon had significant VATS experience prior to transitioning into robotic technology. In aggregate, these studies have focused on surgeons in practice, with suggestion that those with more VATS experience will have a shorter learning curve [8]. Residents are already mandated to learn thoracotomy and VATS by the American Board of Thoracic Surgery and in the UK as per their essential training requirements. Pardolesi *et al.* had performed a comparative study on the learning curves of 2 surgeons for robot-assisted thoracic surgery (RATS) and video-assisted thoracic surgery, which showed the curve for RATS was with 2 reductions in duration of surgery, one after 18 cases and another after 90 cases, and was comparable to the one for VATS [9].

Our study shows that the mixed VATS+RATS group, with VATS as the predominant approach, incorporated into extensive RATS training platform and hence using RATS for independent anatomical lung resection in at least 25% of these centers.

### Robot-assisted thoracic surgery versus video-assisted thoracic surgery

RATS differs from VATS in lung retraction, dissection, carbon dioxide insufflation, camera adjustment, lack of tactile feedback and limited manoeuvrability within the chest [5]. The absence of tactile feedback, further translates into requirement for a different dissection strategy from that of a thoracoscopy [5]. Similarly, another fundamental difference between the dual lens three-dimensional camera and the two-dimensional single lens VATS camera is that VATS cases are done in a broad view for all participants to see. But a robotic approach requires frequent toggling between a broad and a close-up view [5].

A large database compared the results of robotic lobectomies or segmentectomies to thoracotomy or VATS and found statistically significant reductions in mortality, length of stay and overall complications rates in RATS in comparison to thoracotomy but found no difference to VATS [10]. Similarly, another meta-analysis published by Liang *et al.* found lower 30-day mortality and shorter length of stay in the RATS group [11]. Li *et al.* in a large retrospective study comparing VATS and robotics for lobectomies in early-stage lung cancer, using propensity scores match, found better results for RATS with respect to number of lymph nodes retrieved, chest tube duration, volume of chest tube drainage in the 1st post-operative day and length of stay [12]. A recently published study from a propensity-matched analyses reports a shorter operative time, shorter chest tube duration and length of stay by RATS with no difference in complications and mortality compared to VATS [2].

In contrast, Huang *et al.*, in a retrospective analysis of 166 patients, compared VATS and robotic approaches for anatomical lung resections performed by the same surgeon and found a higher rate of prolonged air leak and length of stay in the robotic group [13]. Another series of 163 VATS and 40 robotic lobectomy patients showed comparable outcomes whether performed early or late [14]. In our survey, 62 units adopted RATS for their resections. Their responses regarding patient outcomes are as below:

1. Early chest drain removal—all with RATS approach by 11 units and no difference observed in 3 (1 centre skipped the response).
2. Less postoperative pain and better lymph node harvest reported by 13 units, lesser pain by VATS reported in 9 units.
3. 2 centres adopting all VATS approach, reported shorter hospital stay.
4. Better lymph node harvest was observed by RATS in 16 units.

There was significant overlap in responses on the above patient outcomes amongst VATS and RATS group.

Overall, 52% admitted no difference in chest drain removal timing, 36.6% reported no difference postoperative pain and 49% observed no difference in length of hospital stay. However, 52% preferred RATS for better lymph node harvesting.

Fifty-two percent of all respondents (89) declared that VATS experience was helpful in RATS training. Seventy-one percent of respondents (126) have acknowledged of being proficient in both VATS and RATS and recommend their colleagues, trainees and fellow surgeons to adopt robotics in their future practice of thoracic surgery. The region-wise summary of adoption and preference of type of minimally invasive approach is described in Table 3.

### Limitations

1. The survey shows good number of responses but could be small compared to number of units with members belonging to ESTS. The possibility of positive publication bias cannot be ruled out due to nature of the survey.
2. The basic questionnaire is limited to outline initial information and idea of the situation of RATS/robotic portal lobectomy and its current status in the practice. Further evidences from a larger RCT or a propensity-matched score analyses would be worth considering.
3. It is important to notice that the findings reported in this study reflect the opinions of the respondents and not necessarily best practice. For instance, the majority of surgeons perceive that both VATS and robotic surgery are important for the future practice in our specialty. This obviously reflects an aspirational preference and should take into account financial and resource allocation.
4. At the time of publication of this paper, SP robot had gained significant status in the robotic practice and in European region, apart from a recent CE approved-usage.
5. We also recommend an additional survey for determining in-depth results on surgical outcome of VATS and RATS, i.e. patient factors for better understanding.

### Strengths

The data are obtained from members all over the world, and information obtained is from trainees to surgeons to chief of the unit, hence covering all the spectrum without duplication or repetition. Even though the survey was intended to understand the current trend in practice in thoracic surgery (RATS) within ESTS membership, it has been successful to initiate a formal robotic fellowship program for ESTS members.

## CONCLUSION

This survey concludes that 71% of surgeons would like to adopt robotics in the future but 52% reported that there are no plans to have this procedure implemented at their institutions in the near future. We also detected a large discrepancy adopting RATS in units that have regular access to the robot. This could be better achieved with introduction of robotics in the training curriculum, integration of proctoring, simulation and other skill enhancing exercises. The cost-effectiveness of the robot is a concern and needs attention. Lack of RATS simulator and dual console should also be addressed in order to improve training. After an American nationwide survey, a RATS curriculum has been integrated into existing thoracic surgery residency programmes [15]. However, this is still not considered in the non-American ESTS membership and hence would be desirable to incorporate in future. It is hopeful that the initiation of recent ESTS robotic fellowship programme will help overcoming this problem with favourable results.

## Supplementary Material

ivae031_Supplementary_Data

## Data Availability

The data underlying this article will be shared on reasonable request to the corresponding author.

## Notes

Presented at the ESTS Annual Meeting, Hague, Netherlands, 19–21 June 2022.

## ABBREVIATIONS

ESTS: European Society of Thoracic Surgeons
MITS: Minimally invasive thoracic surgery
RATS: Robotic-assisted thoracic surgery
VATS: Video-assisted thoracoscopic surgery
