# Supplementary material for: Results of an exploratory survey within ESTS membership in 2022 on current trend of robotic-assisted thoracic surgery and its training perspectives
Source: Interdiscip Cardiovasc Thorac Surg. 2024 Mar 5;38(4):ivae031. doi: 10.1093/icvts/ivae031 (PMC11014782; doi:10.1093/icvts/ivae031)
Supplement: ivae031_Supplementary_Data [file ivae031_supplementary_data.zip › Annex 1 - ICVTS.docx]

**Annex 1 - Questionnaire:**

| Who are you? | Trainee in thoracic surgery,  board certified thoracic surgeon,  chief of unit,  other (please specify) |
| --- | --- |
| If you are a certified thoracic surgeon, are you? | Chief of unit,  surgeon in the unit of thoracic surgery, other (please specify) |
| In what country do you work? |  |
| Are you from an academic institution? | Yes  No |
| Has your institution? | 0- no robotic system,  1- no system but making plans to purchase a system,  2- have a system but have limited access to less than 50% of resections,  3- have a system and have good access to it,  4- have a system and also access to robotic proctoring (training) |
| Which robotic system is available in your institution? | None,  Da Vinci Intuitive Si,  Da Vinci Intuitive X,  Da Vinci Intuitive Xi,  other than Da Vinci Intuitive (please specify) |
| Does you robotic system have dual console? | Yes all systems,  yes some systems,  no |
| Is there a RATS simulator available in your center? | Yes  No |
| Are you performing anatomical lung resections independently? | Yes  No |
| If yes, by which approach (multiple answers allowed) | Open  VATS  RATS |
| You can specify the total number of surgical anatomical lung resections cases, respectively, for open / VATS/RATS | Open ……………..  VATS………….…  RATS……………. |
| Approximately, in percentage, how many cases of anatomical lung resections have been performed in your institution in 2019 (pre-covid)? | You can specify the total number of surgical anatomical lung resections cases, respectively, for:  Open  VATS  RATS |
| How many hours did you spend on a training platform already, in total, on – a robotic simulator, RATS wet/dry lab, VATS simulator, VATS wet/dry lab? | None,  some time (<25%),  half of my time (50%),  most of my time (75%),  full immersion (100%) |
| Is robotic surgery included in the curriculum of the structured training platform at your institution for board certification to become a thoracic surgeon? | Yes,  no and no plans to introduce,  no but plans to introduce,  not applicable (e.g.-country has no board certification or thoracic surgery) |
| Do you think that experience in VATS anatomical lung resections is a prerequisite (or help) to start training with a robot? | Yes  No.  If yes, how please specify: |
| Should future thoracic surgeons be proficient in both VATS and RATS anatomical resections? | Yes,  No only by VATS,  No only by RATS,  Other (please specify) |
| In your experience, is there – earlier chest drain removal, less postoperative pain, shorter hospital stay, better lymph node harvest? | VATS,  RATS,  no difference,  don’t know |
| Are you recommending your colleagues, trainees, surgeons to learn/adopt robotics in their future thoracic surgery practice in your center / region? | Yes  No.  If yes, please specify why: |
