# Supplementary material for: Results of an exploratory survey within ESTS membership in 2022 on current trend of robotic-assisted thoracic surgery and its training perspectives
Source: Interdiscip Cardiovasc Thorac Surg. 2024 Mar 5;38(4):ivae031. doi: 10.1093/icvts/ivae031 (PMC11014782; doi:10.1093/icvts/ivae031)
Supplement: ivae031_Supplementary_Data [file ivae031_supplementary_data.zip › Annex 2 - ICVTS.docx]

**Annex 2 – Statistical analysis:**

|  | | Has your institution? | | | | | | | | | | | | | | ***p*** |
| --- | --- | --- | --- | --- | --- | --- | --- | --- | --- | --- | --- | --- | --- | --- | --- | --- |
|  |  | *No robotic system* | *No system but making plans to purchase a system* | | | | *Have a system but have limited access to less than 50% of resections* | | | | | | *Have a system and have good access to it* | | *Have a system and also access to robotic proctoring (training)* |  |
| Are you from an academic institution? | **No** | 15  (37.5%) | 4  (10%) | | | | 9  (22.5%) | | | | | | 5  (12.5%) | | 7  (17.5%) | 0.08 |
|  | **Yes** | 23  (18%) | 10  (7.8%) | | | | 43  (33.6%) | | | | | | 30  (23.4%) | | 22  (17.2%) |  |
|  | | Which robotic system is available in your institution? | | | | | | | | | | | | | | 0.10 |
|  |  | *None* | *Da Vinci Intuitive Model Xi* | | | | *Da Vinci Intuitive Model X* | | | | | | *Da Vinci Intuitive Model Si* | | *Other than Da Vinci Intuitive* |  |
| Are you from an academic institution? | **No** | 18  (45%) | 16  (40%) | | | | 3  (7.5%) | | | | | | 3  (7.5%) | | 0  - |  |
|  | **Yes** | 30  (23.4%) | 78  (60.9%) | | | | 10  (7.8%) | | | | | | 9  (7%) | | 1  (0.8%) |  |
|  |  | Does your robotic system(s) have dual console? | | | | | | | | | | | | | | **0.002** |
|  |  | *Yes all systems* | | | *Yes some systems* | | | | | | | *No* | | | |  |
| Are you from an academic institution? | **No** | 3  (7.9%) | | | 2  (5.3%) | | | | | | | 33  (86.8%) | | | |  |
|  | **Yes** | 68  (55.7%) | | | 38  (31.1%) | | | | | | | 16  (13.1%) | | | |  |
|  | | Is there a RATS simulator available in your centre? | | | | | | | | | | | | | | **0.006** |
|  |  | *No* | | | | | | | | | | | | *Yes* | |  |
| Are you from an academic institution? | **No** | 27  (69.2%) | | | | | | | | | | | | 12  (30.8%) | |  |
|  | **Yes** | 55  (43.3%) | | | | | | | | | | | | 72  (56.7%) | |  |
|  |  | Use of open approach | | | | | | | | | | | | | | 0.25 |
|  |  | *No* | | | | | | | | | | | | *Yes* | |  |
| Are you from an academic institution? | **No** | 10  (25%) | | | | | | | | | | | | 30  (75%) | |  |
|  | **Yes** | 46  (35.9%) | | | | | | | | | | | | 82  (64.1%) | |  |
|  |  | Use of VATS approach | | | | | | | | | | | | | | 0.25 |
|  |  | *No* | | | | | | | | | | | | *Yes* | |  |
| Are you from an academic institution? | **No** | 5  (12.5%) | | | | | | | | | | | | 35  (87.5%) | |  |
|  | **Yes** | 28  (21.9%) | | | | | | | | | | | | 100  (78.1%) | |  |
|  |  | Use of RATS approach | | | | | | | | | | | | | | **-** |
|  |  | *No* | | | | | | | | | | | | *Yes* | |  |
| Are you from an academic institution? | **No** | - | | | | | | | | | | | | 12  (100%) | |  |
|  | **Yes** | - | | | | | | | | | | | | 47  (100%) | |  |
|  |  | Is robotic surgery included in the curriculum of the structured training program at your institution for board certification to become a Thoracic Surgeon? | | | | | | | | | | | | | | 0.98 |
|  |  | *Not applicable (e.g., country has no Board certification or Thoracic surgery)* | | | *No and no plans to introduce* | | | | | | | *No, but plans to introduce* | | | *Yes* |  |
| Are you from an academic institution? | **No** | 2  (5%) | | | 21  (52.5%) | | | | | | | 14  (35%) | | | 3  (7.5%) |  |
|  | **Yes** | 5  (3.9%) | | | 68  (53.1%) | | | | | | | 47  (36.7%) | | | 8  (6.3%) |  |
|  |  | Do you think that experience in VATS anatomical lung resections is a prerequisite (or help) to start training with a robot? | | | | | | | | | | | | | | 0.71 |
|  |  | *No* | | | | | | *Yes* | | | | | | | |  |
| Are you from an academic institution? | **No** | 16  (40%) | | | | | | 24  (60%) | | | | | | | |  |
|  | **Yes** | 56  (43.8%) | | | | | | 72  (56.3%) | | | | | | | |  |
|  |  | Should future thoracic surgeons be proficient in both VATS and RATS anatomical resections? | | | | | | | | | | | | | | 0.70 |
|  |  | *Yes* | | | *No, only by VATS* | | | | | | | *No, only by RATS* | | *Other* | |  |
| Are you from an academic institution | **No** | 29  (72.5%) | | | 7  (17.5%) | | | | | | | 1  (2.5%) | | 3  (7.5%) | |  |
|  | **Yes** | 90  (70.3%) | | | 27  (21.1%) | | | | | | | 6  (4.7%) | | 5  (3.9%) | |  |
|  |  | In your experience, is there – earlier chest drain removal, less postoperative pain, shorter hospital stay, better lymph node harvest? | | | | | | | | | | | | | | 0.40 |
|  |  | *Don’t know* | | *No difference* | | | | | | | *VATS* | | | *RATS* | |  |
| Are you from an academic institution | **No** | 13  (32.5%) | | 20  (50%) | | | | | | | 4  (10%) | | | 3  (7.5%) | |  |
|  | **Yes** | 28  (22%) | | 67  (52.8%) | | | | | | | 12  (9.4%) | | | 20  (15.7%) | |  |
|  |  | Are you recommending your colleagues, trainees, surgeons to learn/adopt robotics in their future thoracic surgery practice in your centre / region? | | | | | | | | | | | | | | 0.05 |
|  |  | *No* | | | | | | | | | | | | *Yes* | |  |
| Are you from an academic institution | **No** | 11  (27.5%) | | | | | | | | | | | | 29  (72.5%) | |  |
|  | **Yes** | 18  (14.1%) | | | | | | | | | | | | 110  (85.9%) | |  |
|  |  | Approximately, in percentage, how many cases of anatomical lung resections have been performed in your institution in 2019 (pre-covid)?  You can specify the total number of surgical anatomical lung resections cases, respectively, for: | | | | | | | | | | | | | | |
|  |  | *Open* | | | | *VATS* | | | | *RATS* | | | | | | |
| Are you from an academic institution | **No** | 37 | | | | 38 | | | | 12 | | | | | | |
|  | **Yes** | 121 | | | | 114 | | | | 65 | | | | | | |
|  | ***p*** | 0.96 | | | | 0.18 | | | | 0.67 | | | | | | |
|  | | How many hours did you spend on a training platform already, in total, on – a robotic simulator, RATS wet/dry lab, VATS simulator, VATS wet/dry lab?  None, some time (<25%), half of my time (50%), most of my time (75%), full immersion (100%) | | | | | | | | | | | | | | |
|  |  | *Robotic*  *Simulator* | | | *RATS Wet/Dry Lab.* | | | | *VATS Simulator* | | | | | *VATS Wet/Dry Lab.* | | |
| Are you from an academic institution | **No** | 35 | | | 35 | | | | 34 | | | | | 37 | | |
|  | **Yes** | 119 | | | 112 | | | | 110 | | | | | 113 | | |
|  | ***p*** | 0.50 | | | 0.11 | | | | 0.98 | | | | | 0.26 | | |
|  |  | How many surgeons (number) at your unit are performing independently RATS | | | | | | | | | | | | | | ***p***  **0.009** |
| Are you from an academic institution | **No** | 40 | | | | | | | | | | | | | |  |
|  | **Yes** | 125 | | | | | | | | | | | | | |  |
